# Binding epitope for recognition of human TRPM4 channel by monoclonal antibody M4M

**DOI:** 10.1038/s41598-022-22077-4

**Published:** 2022-11-15

**Authors:** Shunhui Wei, Julian Behn, Charlene Priscilla Poore, See Wee Low, Bernd Nilius, Hao Fan, Ping Liao

**Affiliations:** 1 Calcium Signalling Laboratory, Department of Research, National Neuroscience Institute, 11 Jalan Tan Tock Seng, Singapore, 308433 Singapore; 2 Bioinformatics Institute (BII), Agency for Science, Technology and Research (A*STAR), Singapore, 138671 Singapore; 3 Yong Loo Lin School of Medicine, National University of Singapore, Singapore, Singapore; 4 Department of Cellular and Molecular Medicine, KU Leuven, Leuven, Belgium; 5 Health and Social Sciences, Singapore Institute of Technology, Singapore, Singapore; 6 Duke-NUS Medical School, Singapore, Singapore

**Keywords:** Structure determination, Microarrays, Ion channels in the nervous system

## Abstract

Mouse monoclonal antibody M4M was recently designed to block human TRPM4 channel. The polypeptide for generating M4M is composed of peptide A1 between the transmembrane segment 5 (S5) and the pore, and a second peptide A2 between the pore and the transmembrane segment 6 (S6). Using peptide microarray, a 4-amino acid sequence EPGF within the A2 was identified to be the binding epitope for M4M. Substitution of EPGF with other amino acids greatly reduced binding affinity. Structural analysis of human TRPM4 structure indicates that EPGF is located externally to the channel pore. A1 is close to the EPGF binding epitope in space, albeit separated by a 37-amino acid peptide. Electrophysiological study reveals that M4M could block human TRPM4, but with no effect on rodent TRPM4 which shares a different amino acid sequence ERGS for the binding motif. Our results demonstrate that M4M is a specific inhibitor for human TRPM4.

## Introduction

Transient receptor potential melastatin member 4 (TRPM4) is a voltage-dependent, non-selective monovalent cation channel^1^. Recently, TRPM4 has been found to play a key role in many diseases. TRPM4 mutations are known to cause cardiac conduction problems such as right bundle-branch block, tachycardia, and Brugada syndrome^2^. In cancers from prostate, liver, urinary bladder, cervix, colon, and large B cell, aberrant TRPM4 activity affects cancer cell growth and migration^3^. In nervous system, TRPM4 plays an important role in neurovascular cell death in diseases such as multiple sclerosis^4^, stroke^5^, head injury^6^, and spinal cord injury^7^. Under ischemia, elevated cytosolic Ca^2+^ level and ATP depletion greatly enhance TRPM4 activity. TRPM4 expression was found upregulated in neurological diseases with hypoxic insult. In together with an increased channel activity, an excessive Na^+^ influx via TRPM4 channel causes oncotic cell death in neurons and vascular endothelial cells^7,8^.

Antidiabetic drug glibenclamide has been reported to inhibit TRPM4 function and is currently under investigation in clinical trials for stroke^9^. Glibenclamide is a widely prescribed antidiabetic medicine for treating type 2 diabetes. It regulates blood glucose level by inhibiting K_ATP_ channels via interacting with SUR1 subunit in pancreatic beta-cells^10^. As TRPM4 forms a channel complex with SUR1, glibenclamide exhibits an inhibitory effect on TRPM4 when SUR1 and TRPM4 are co-expressed at a certain ratio^11^. Without SUR1, glibenclamide has no effect on TRPM4 activity^12^. During the past years, we seek to inhibit TRPM4 function by targeting TRPM4 directly. siRNA was first used to suppress TRPM4 expression in both in vitro and in vivo studies. Following stroke induction, application of TRPM4 siRNA could protect neurons and vascular endothelial cells. Accordingly, functional improvement was identified in both permanent and transient stroke models^13,14^. As siRNA acts on transcriptional level, TRPM4 siRNA needs to be applied at very early stage of disease onset, prior to TRPM4 protein upregulation. Considering the limitation of siRNA, we seek to develop a specific blocker, directly acting on the TRPM4 protein.

Around 12 years ago, we started to generate a polyclonal antibody M4P against a 28-amino acid polypeptide between the transmembrane segment 5 (S5) and 6 (S6) of rat TRPM4 channel^15^. M4P was shown to reduce TRPM4 current under both normoxic and hypoxic conditions, protecting vascular endothelial cells and neurons from oncotic cell death following hypoxia^16^. Application of M4P ameliorated reperfusion injury in an animal model of stroke^15^. Based on the success of M4P, we further developed a monoclonal antibody M4M against human TRPM4^17^. The polypeptide targeted by M4M is a 21-amino acid sequence between S5 and S6 of human TRPM4 channel. This polypeptide is composed of two small peptides separated by the hydrophobic channel pore^18^. It is unclear what is the binding epitope, and how the location of individual amino acid affects M4M action. In this study, we performed epitope mapping, amino acid substitution, structural analysis and electrophysiological study to examine the binding epitope for M4M, and the specificity of M4M to human TRPM4 channel.

## Result

### Identification of binding epitope for M4M

**Figure 1:**
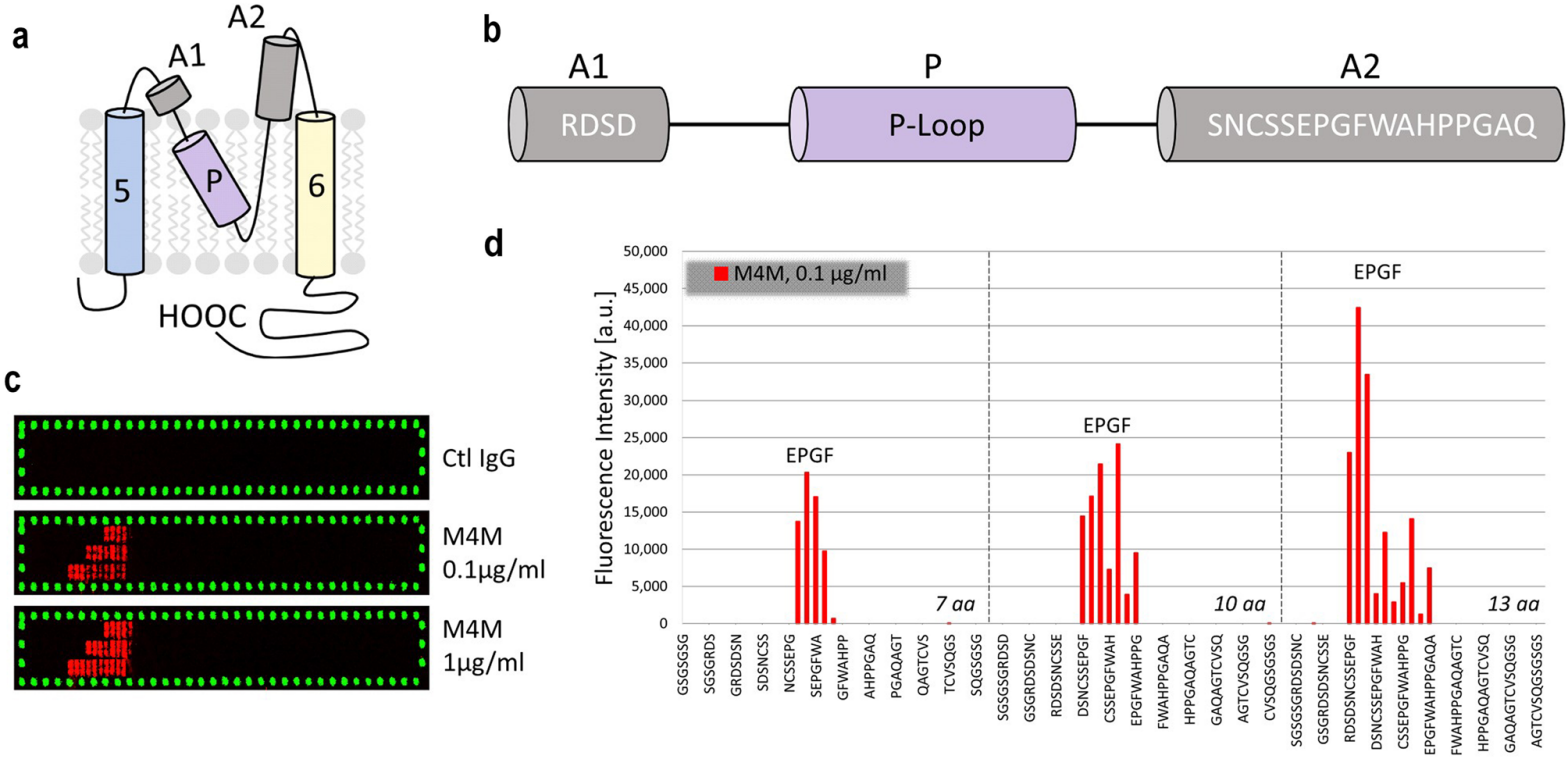
Identification of binding epitope for M4M. (**a**) Schematic representation of the transmembrane segments 5 and 6 of the human TRPM4 channel. Antigenic epitope for M4P is labelled as A1 and A2. P: pore-loop. (**b**) Amino acid sequence of A1 and A2, separated by channel pore (p-loop). (**c**) Read-outs of a conformational peptide microarray incubated with control mouse IgG of 1 µg/ml or M4M at a concentration of 0.1 and 1 µg/ml, followed by staining with the secondary antibodies (red: sample staining; green: control staining). For each read-out picture, top row: 7 amino-acid fragments; middle row: 10 amino-acid fragments; bottom row: 13 amino-acid fragments. (**d**) At peptide lengths of 7, 10, and 13 amino acids, a very strong antibody response against epitope-like spot patterns formed by adjacent peptides with the consensus motif EPGF.

Mouse monoclonal antibody M4M was designed to target an extracellular polypeptide of human TRPM4 (Fig. 1a). This polypeptide is composed of two antigenic sequences A1 and A2 which is separated by the channel pore-forming epitope, P-loop. A1 contains 4 amino acids and A2 contains 17 amino acids (Fig. 1b). To identify the binding epitope for M4M, peptide microarrays were performed on the antigenic polypeptide RDSDSNCSSEPGFWAHPPGAQ (Fig. 1c). The peptide together with linkers was converted into 7, 10 and 13 amino acid peptides with overlaps of 6, 9 and 12 amino acids. Incubation with M4M at various concentrations ranging from 0.1 to 100 µg/ml demonstrated a similar staining pattern (Fig. 1c) with high signal-to-noise ratios. Control IgG showed no binding to the antigenic polypeptide. The strong antibody response observed in all 7, 10 and 13 amino acid peptides revealed that a 4-amino acid sequence EPGF is the binding epitope for M4M (Fig. 1d).

### EPGF is critical for M4M binding

**Figure 2:**
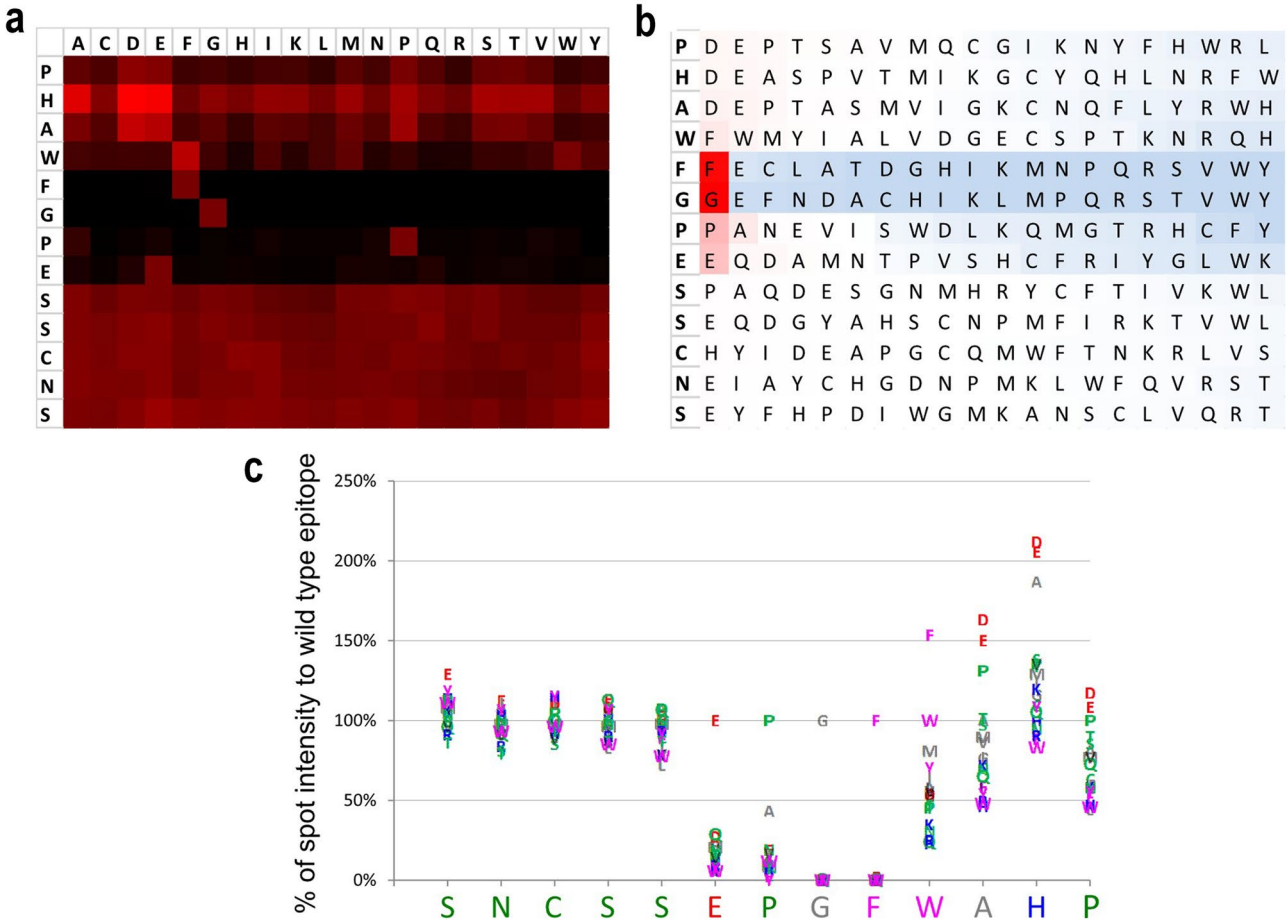
Epitope substitution assay. The heat map (**a**), substitution matrix (**b**) and amino acid plot (**c**) of mouse antibody M4M assayed against the substitution scan of cyclic constrained wild-type peptide ^1^SNCSSEPGFWAHP^13^.

The binding epitope EPGF and adjacent amino acids ^1^SNCSSEPGFWAHP^13^ was further examined by substitution of individual amino acid for the 20 standard amino acids. The heat map, the substitution matrix and the amino acid plot (Fig. 2a–c) highlighted a conserved core motif ^6^EPGF^9^ framed by N- and C-terminal variable stretches ^1^SNCSS^5^ and ^10^WAHP^13^.

Amino acid positions ^8^G and F^9^ exhibited an essential character, exchange for any other amino acid resulted in a complete loss of antibody binding (Fig. 2c). Amino acid position ^6^E was strongly conserved and showed at least 72% lower signal intensities after exchange for any other amino acid. Amino acid position ^7^P exhibited also a strongly conserved character, exchange for A reduced the signal intensity by 57%, while any other substitution resulted in at least 81% lower signal intensities compared to the wild-type amino acid.

All other amino acid positions of the N- and C-terminal variable stretches ^1^SNCSS^5^ and ^10^WAHP^13^ exhibited a variable character without preference for the wild-type amino acids. Despite its predominantly variable character, amino acid position ^10^ W exhibited a minor preference for aromatic amino acids F, W and Y.

### M4M is specific for human TRPM4

**Figure 3:**
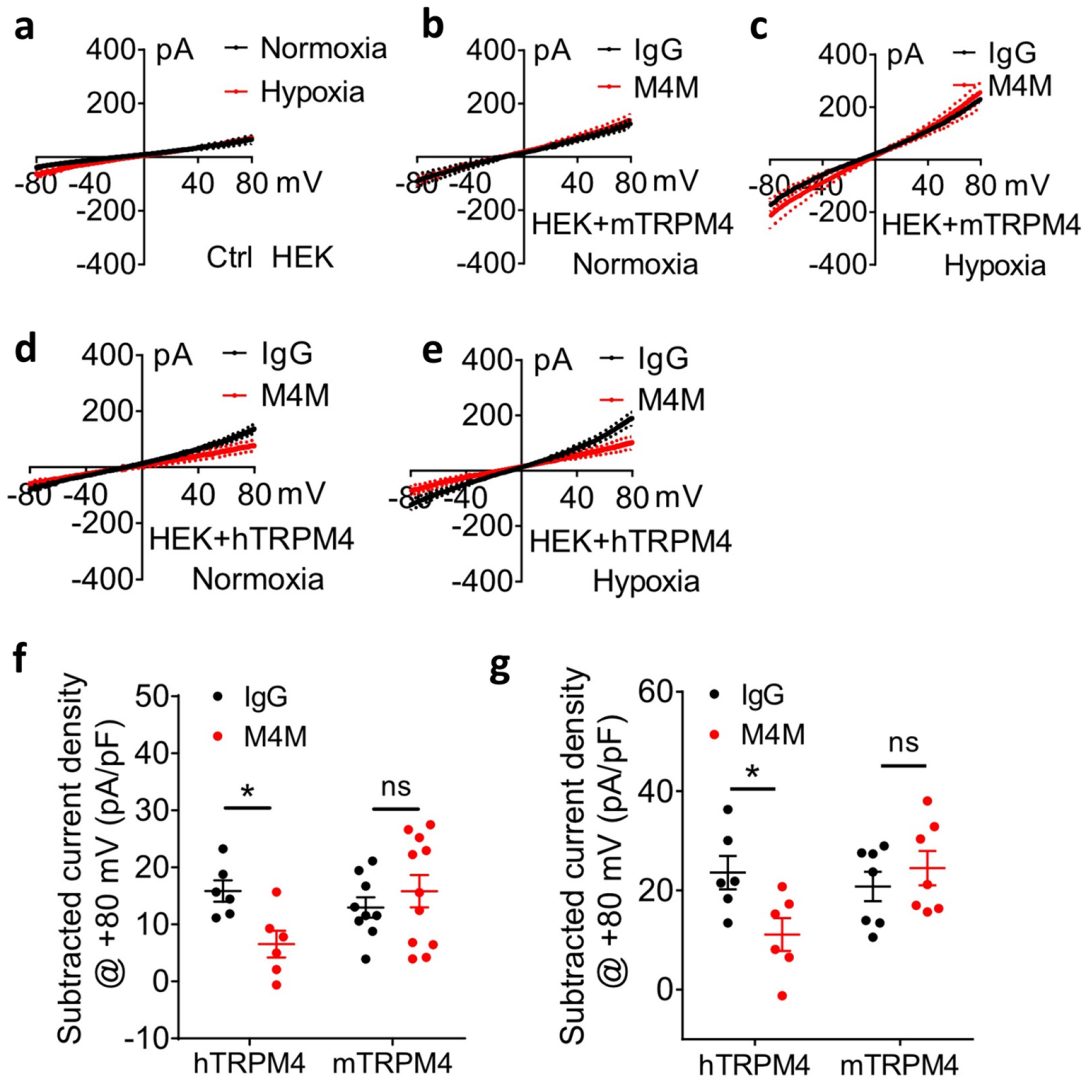
Electrophysiological characterization of M4M. (**a**) The current voltage relationships were obtained from non-transfected HEK293 cells under normoxia and 7-min hypoxia. Ramp protocols were applied from − 80 to + 80 mV with a holding potential at 0 mV. n = 8 cells. (**b**) The current voltage relationships from HEK293 cells transfected with mouse TRPM4 under normoxia. The cells were pretreated with control IgG (n = 8 cells) or M4M (n = 11 cells) at 20.8 μg/ml for 30 min before patch clamping. (**c**) Comparison of current voltage relationships between IgG (n = 8 cells) and M4M (n = 10 cells) treated HEK293 cells under hypoxia with mouse TRPM4 overexpression. (**d**) Comparison of current voltage relationships between IgG and M4M treated HEK293 cells under normoxia with human TRPM4 overexpression. n = 6 cells. (**e**) Comparison of current voltage relationships between IgG and M4M treated HEK293 cells under hypoxia with human TRPM4 overexpression. n = 6 cells. (**f**) Summary of TRPM4 current density at + 80 mV under normoxia. Background currents from (**a**) were subtracted. hTRPM4: transfection with human TRPM4; mTRPM4: transfection with mouse TRPM4. (**g**) Summary of subtracted current density at + 80 mV under hypoxia. *p < 0.05, student’s *t* test.

To functionally characterize M4M, human or mouse TRPM4 was transiently expressed in HEK293 cells. TRPM4 channel is activated by incubation under hypoxia for 7 min. In control non-transfected HEK293 cells, baseline currents were observed by a ramp protocol from − 80 mV to + 80 mV (Fig. 3a). When the mouse TRPM4 was transfected, a larger current was observed in both IgG and M4M treated cells comparing to non-transfected cells (Fig. 3b). No difference was found between IgG and M4M treatments. We next activated mouse TRPM4 by 7-min hypoxic induction. Comparing to normoxic condition in Fig. 3b, hypoxia induced a larger current in both IgG and M4M treatments (Fig. 3c). Again, no difference was identified between the two groups. When human TRPM4 was introduced into HEK293 cells, under normoxic condition, IgG treatment yields a larger current than M4M treatment (Fig. 3d). After 7-min hypoxic induction, currents observed under M4M treatment is still smaller than IgG treatment (Fig. 3e).

We further analyzed the current density at + 80 mV after subtracting the baseline current. Under normoxic condition, human TRPM4 current was inhibited by M4M treatment. In contrast, both IgG and M4M treatments have a similar mouse TRPM4 current (Fig. 3f). After 7-min hypoxic induction, human TRPM4 current was again inhibited by M4M, and no difference was identified between IgG and M4M treatment in mouse TRPM4 expressed cells (Fig. 3g). These results indicate that M4M can effectively inhibit human TRPM4 current under both normoxic and hypoxic conditions, whereas M4M has no effect on mouse TRPM4 current.

**Figure 4:**
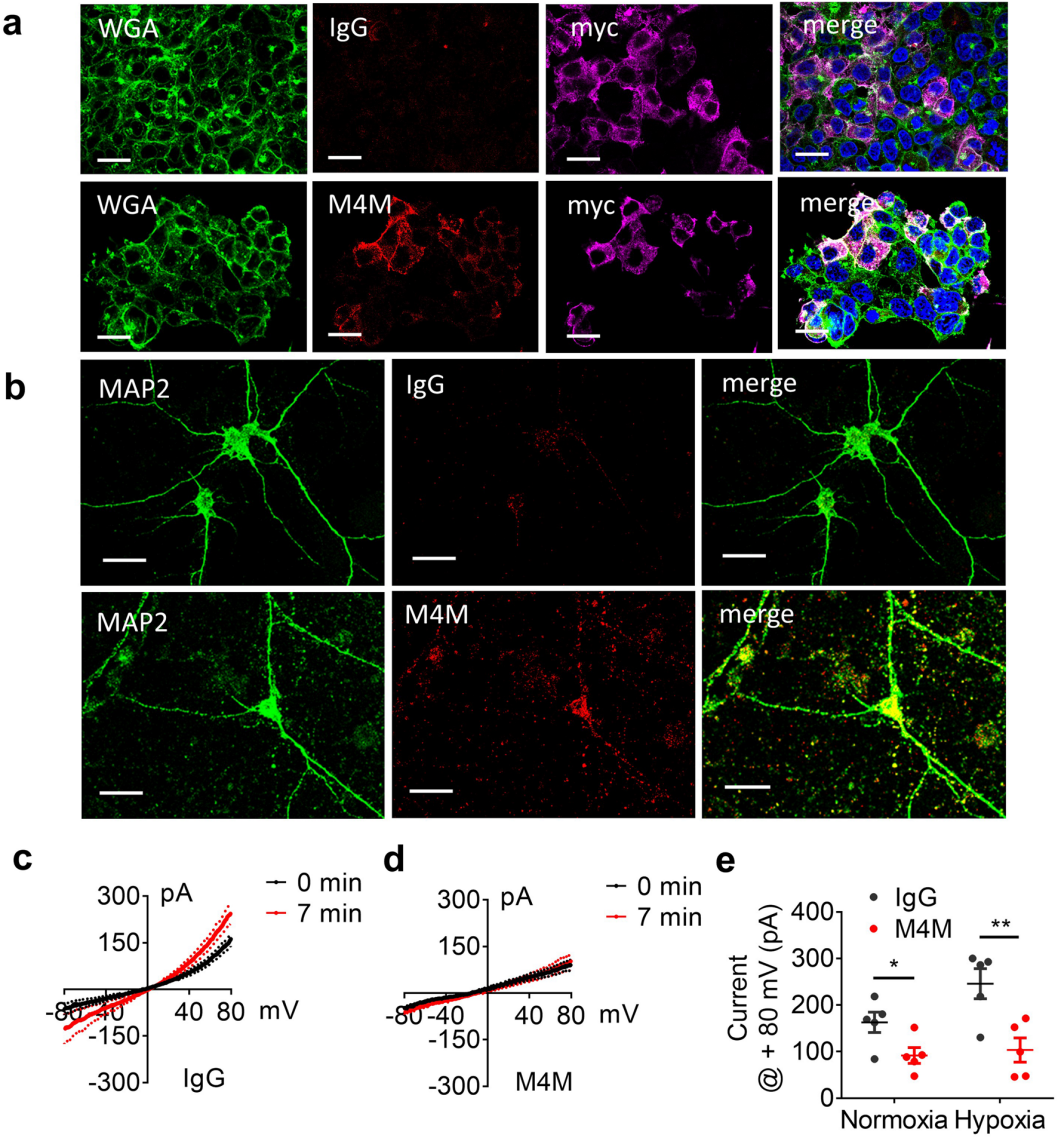
M4M detecting of hTRPM4 and its effect on neurons from transgenic rat carrying human TRPM4 sequence. (**a**) Immunofluorescence staining of HEK transfected with Myc-DDK-tagged human TRPM4 detected using IgG (upper panel) or M4M (lower panel). WGA, cell surface marker (green color) and human IgG or M4M staining (red color), Myc staining for hTRPM4 (pink color). Scale bars: 20 µm. (**b**) After 24 h OGD, human IgG (upper panel) or M4M staining (lower panel, red color) on neurons from transgenic rat carrying human TRPM4 sequence. MAP2, neuron marker (green color). Scale bars: 20 µm. (**c**) Comparison of current voltage relationships between normoxia and hypoxia neurons treated with IgG, n = 5 cells. (**d**) Comparison of current voltage relationships between normoxia and hypoxia neurons treated with M4M, n = 5 cells. (**e**) Summary of TRPM4 current at + 80 mV under normoxia and hypoxia treated with IgG or M4M (right). *p < 0.05, **p < 0.01, student’s t test.

To examine whether M4M could bind TRPM4 channel on the surface of live cells, HEK 293 cells transfected with hTRPM4 were cultured, the cells were then fixed and human IgG or M4M were used (Fig. 4a), M4M could be identified on the surface of HEK 293 cells with the cell surface marker WGA. No surface staining was observed in cells incubated with control IgG. M4M also detected hTRPM4 on primary cultured neurons from transgenic rat carrying human TRPM4 sequence. No signal was detected by control IgG (Fig. 4b). Electrophysiology study identified a hypoxia-induced current increase in control IgG treated neurons. Whereas M4M incubation successfully blocked hypoxia-induced current increase (Fig. 4c–e). These results demonstrate that replacement of the binding site with human TRPM4 sequence renders rat TRPM4 sensitive to M4M blockade.

### Structural analysis

**Figure 5:**
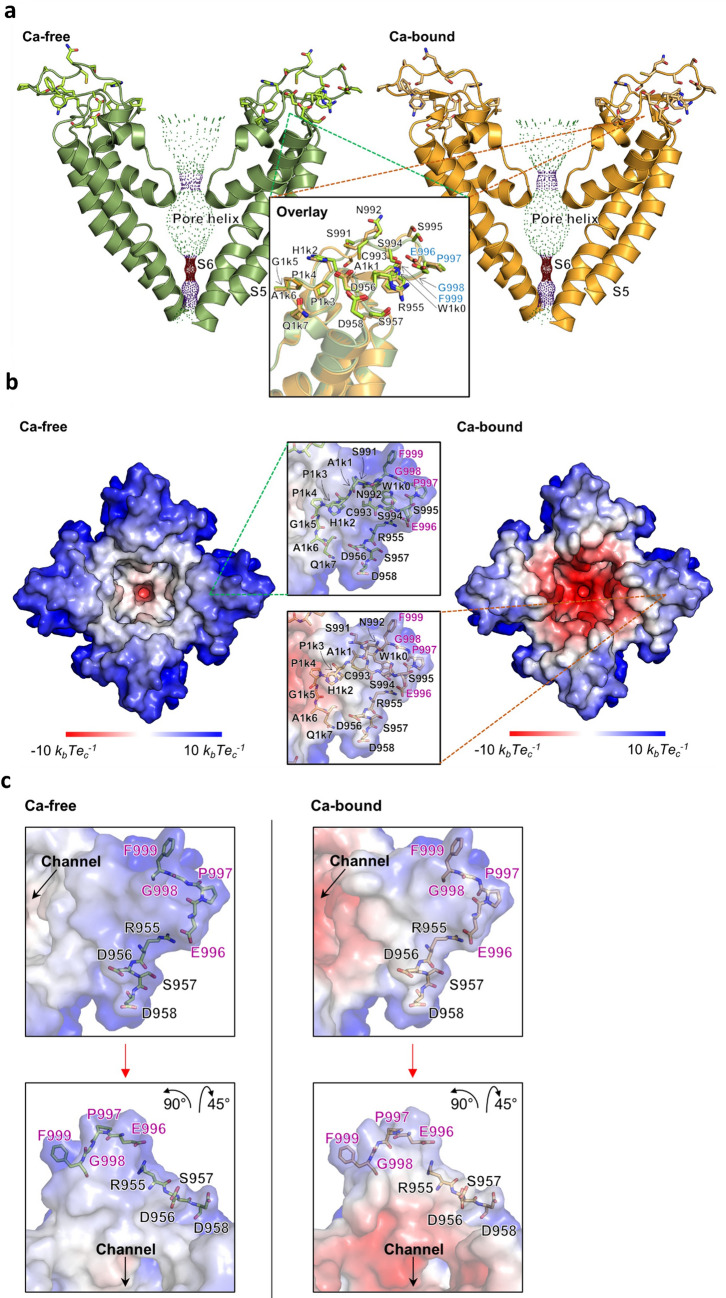
Human TRPM4 channel pore structure. (**a**) Human TRPM4 channel pore structure in its calcium-free (left, in green) and calcium-bound (right, in orange) closed states, visualized for the S5—pore helix—S6 region without the front and rear tetramer units. The 21 amino acid peptide sequence residues are shown in stick representation. Close-up window depicts a superposition of the peptide binding region with labelled residues. The proposed EPGF binding motif residues are highlighted in blue. The channel pore is visualized in dots representation. For clearness, residue labels X100n with n = 0, 1,…,9 are abbreviated as X1kn. Channel pore radii for dots visualization were calculated using the program HOLE (HOLE release 2.2.005, 07 August 2016. http://www.holeprogram.org/). **(b)** Channel entrance view of the human TRPM4 tetramer in 2 states. Solvent-excluded surface colored by electrostatic potential is shown for the calcium-free (left) and calcium-bound (right) closed states, visualized for the S5—pore helix—S6 region. Close-up windows show the 21 amino acid peptide sequence residues for the calcium-free (in green sticks) and calcium-bound (in orange sticks) closed states. Residues are labelled and the proposed EPGF binding motif residues are highlighted in magenta. Colored scale bar indicates the surface electrostatic potentials. For clearness, residue labels X100n with n = 0, 1,…,9 are abbreviated as X1kn. Electrostatic surface potentials were calculated with the APBS (Version 3.0, https://www.poissonboltzmann.org/). (**c**) Close-up windows from 2 different angles showing the proposed EPGF binding motif residues as well as residues R955-D958 for the calcium-free (left, in green sticks) and calcium-bound (right, in orange sticks) human TRPM4 closed states, visualized for the S5—pore helix—S6 region from channel entrance perspective. Residues are labelled and the proposed EPGF binding motif residues are highlighted in magenta. Channel pores, electrostatic surfaces and protein structures were visualized using the PyMOL (Molecular Graphics System, Version 1.8. 2015. Schrodinger, LLC https://pymol.org/).

TRPM4 is a calcium-activated channel; based on the recently published human TRPM4 structure^19^, location of antigenic sequence within the central pore region of human TRPM4 was investigated under calcium-free and calcium-bound conditions (Fig. 5a). This region contains S5, S6, and pore-loop, forming the ion-permeation pathway. Both A1 and A2 sequences are located extracellularly, encircling the vestibule at the external entrance of the channel. A2 forms part of the exceptionally long loop between the filter and S6. Overlay of both calcium-free and calcium-bound structures identifies no major changes to the positions of A1 and A2.

Highly negative electrostatic surface potential was measured within the inner surface of the vestibule under calcium-bound condition (Fig. 5b, channel entrance view). Again, both calcium-free and calcium-bound structures exhibit similar extracellular formations. Binding epitope EPGF is located to the outermost of the channel, forming the tip of the extracellular turret regardless of calcium presence. A1 peptide RDSD, although separated from A2 by the pore, is close to EPGF epitope (Fig. 5c). N-linked glycosylation site N992 is located on the other side to EPGF, near the channel entrance (Fig. 5a,b).

## Discussion

More than 20 years ago, a TRPC1 antibody has been developed by targeting an extracellular domain to inhibit the channel activity^20^. More antibodies were subsequently reported for blocking other TRP channels^21^. These antibodies target the 3rd extracellular domain (E3) between the transmembrane segment 5 (S5) and the pore of the channels. When we started to develop TRPM4 blocking antibody^22^, we found that the E3 domain of TRPM4 is short and contains mostly hydrophobic amino acids. Therefore, a longer sequence covering the whole sequence linking S5 and S6 was taken into consideration for designing the antigen. The first TRPM4 blocking antibody that we developed is a polyclonal antibody against the rat TRPM4 channel^15^. Recently, we produced a monoclonal antibody M4M targeting a similar peptide of human TRPM4^17^. M4M was revealed to bind to human TRPM4 on the surface of cell membrane. Electrophysiological study further proved that M4M could inhibit human TRPM4 activity.

The antigenic sequence for producing M4M contains an A1 peptide of 4-amino acid sequence RDSD, and an A2 peptide of 17-amino acid sequence SNCSSEPGFWAHPPGAQ from human TRPM4 channel (Fig. 1). The selection of A1 epitope RDSD was based on hydrophobicity analysis. Majority of the remaining sequences within the E3 region are hydrophobic, with less accessibility for antibody binding. In contrast, the sequence linking the pore and S6, where A2 is located, is long and contains mostly hydrophilic amino acids. The joining of A1 and A2 into one polypeptide to produce M4M is an unprecedented attempt as the two regions are separated by a rather long pore-spanning 32-amino acid peptide. Our hypothesis is that A1 and A2, albeit separated by the p-loop, are close in space, contributing to antibody recognition. When M4M was successfully generated, one question arises: where is the binding site? The traditional E3 region (A1 sequence), or the new A2 region after the pore?

In this study, robust evidence from epitope mapping and subsequent substitution assay clearly shows that the 4-amino acid epitope EPGF within the A2 region is the binding epitope for M4M. EPGF lies in the middle of A2, away from the channel pore by 5 amino acids. Based on the published structure of human TRPM4^19^, structural analysis of human TRPM4 reveals that EPGF epitope is located outermost, protruding into the extracellular space, making it ideal for antibody binding. The A1 sequence RDSD is also located outside the channel, but closer to the pore (Fig. 5c). As hypothesized, albeit being separated by a lengthy 37-amino acid peptide, A1 and EPGF are physically close in the space. Although A1 is not part of the binding epitope, it may provide a support to stabilize the antibody binding. Further study is required to clarify the exact role of A1 in M4M function. It should be noted that A2 contains a glycosylation site Asn992 with the attached glycan pointing toward the extracellular space^19^. Asn992 is close to the channel pore and seems not to interfere with M4M binding.

As a Ca^2+^-activated cation channel, binding of Ca^2+^ to TRPM4 is hypothesized to precede voltage-dependent opening^23^. The Ca^2+^ binding site is located in a hydrophilic pocket within the cytoplasmic side of the S1-S4 domains^19^. Here, we found that the presence of calcium does not change the position of TRPM4 structure, including the EPGF binding epitope. Thus M4M could bind to both Ca^2+^-free and Ca^2+^-bound TRPM4 channels. How M4M binding affects Ca^2+^ sensitivity remains unknown. As the M4M binding site is at the extracellular side of the channel and the Ca^2+^ binding site is at the cytoplasmic side, M4M is unlikely to affect Ca^2+^ binding directly. The blocking effect of M4M is rather from a direct blockade on the channel pore or via inhibiting conformational change during channel activation. Hypoxia is known to increase intracellular calcium level which activates TRPM4 channel^24^. Our result indicates that M4M could bind to human TRPM4 under both resting and activated states. This is consistent with our previous study showing that M4M could inhibit human TRPM4 under both normoxic and hypoxic conditions^17^. Such property of M4M has clinical relevance as the tissue under disease attack usually contains a mixture of healthy cells as well as cells affected by hypoxia. Inhibition of TRPM4 current in hypoxic cells could alleviate oncotic cell death, whereas in normoxic cells, binding of M4M prevents potential oncosis when hypoxia spreads to those cells.

Substitution assay revealed that each amino acid of the EPGF epitope is critical for binding. Exchanging to any other amino acid greatly reduced binding affinity. Comparing to human TRPM4 sequence of EPGF, mouse TRPM4 channel contains ERGS in the corresponding positions. Our electrophysiological study shows that M4M has no effect on mouse TRPM4, confirming the specificity of M4M to human TRPM4.

Peptide-based epitope mapping is ideal for linear and relatively simple conformational epitopes. Complex epitopes with tertiary and/or quaternary structure may not be well characterized^25^. For TRPM4, four subunits are required to form a functional channel^24^. To characterize that the extracellular domain of human TRPM4 is critical for M4M binding, extracellular domain between S5 and 6 of rat TRPM4 was replaced with the corresponding human sequence. Immunostaining and patch clamp study confirm that this mutant rat TRPM4 is functional and becomes sensitive to M4M blockade. This result further proves that M4M is specific for human TRPM4.

Current TRPM4 blockers 9-phenanthrol and glibenclamide have various challenges of non-specificity, toxicity or requiring the presence of additional subunits^13^. TRPM4 blocking antibody demonstrates the advantage of strong specificity. Our previous studies on polyclonal antibody M4P have shown the capability of TRPM4 blocking antibody to inhibit TRPM4 functions, both in vitro and in vivo^15,16^. The production of monoclonal antibody M4M marks an advancement of our attempt to develop a novel therapy for neurological diseases relating to TRPM4 abnormal functions^26^. Identification of the extracellular binding epitope indicates that TRPM4 blocking antibody could gain access to the channel easily from outside of the cell. It should be noted that M4M is a mouse monoclonal antibody which could generate allogenic immune responses if used in patients. Humanization of M4M is the next step to reduce immune rejection.

## Materials and methods

### Production of monoclonal antibody M4M

The production of mouse monoclonal antibody M4M has been described previously^17^. A KLH conjugated 21-amino acid antigenic polypeptide (RDSDSNCSSEPGFWAHPPGAQ) which is close to the channel pore of human TRPM4 was used for antibody production. Among the several clones produced, M4M was selected to own the best binding affinity and inhibitory effect on TRPM4 channel activity.

### Epitope mapping

Peptide microarrays were performed by PEPperPRINT (Heidelberg, Germany). The sequence of peptide RDSDSNCSSEPGFWAHPPGAQ was elongated with neutral GSGSGSG linkers at the C- and N-terminus to avoid truncated peptides. The elongated peptide sequence was converted into 7, 10 and 13 amino acid peptides with peptide-peptide overlaps of 6, 9 and 12 amino acids. After on-chip peptide synthesis, all peptides were cyclized via a thioether linkage between a C-terminal cysteine and an appropriately modified N-terminus. The resulting conformational peptide microarrays contained 99 different peptides printed in duplicate (198 peptide spots) and were framed by additional HA (YPYDVPDYAG, 74 spots) control peptides. The corresponding peptide microarrays were incubated with M4M at a concentration of 0.1 μg/ml in incubation buffer, followed by staining with the secondary goat anti-mouse IgG (H+L) DyLight680 antibody (0.2 µg/ml, Thermo Fisher). The control antibody is mouse monoclonal anti-HA (12CA5) DyLight800 (0.2 µg/ml). Read-out was performed with an Innopsys InnoScan 710-IR Microarray Scanner at scanning gains of 50/10 (red/green). The additional HA control peptides framing the peptide microarrays were subsequently stained with the control antibody as internal quality control to confirm assay performance and peptide microarray integrity. Microarray image analysis was done with PepSlide Analyzer (Sicasys GmbH).

### Generation of a transgenic rat carrying human TRPM4 sequence

Using CRISPR/Cas9 technology, we generated a transgenic rat model carrying human TRPM4 sequence. The binding motif, which is located between exon 19 and 20, was replaced with the corresponding human TRPM4 sequence (mutation sequence is from rat QDRSLPSILRRVFYRPYLQIFGQIPQEEMDVALMNPSNCSAERGSWAHPEGPV to human RDSDFPSILRRVFYRPYLQIFGQIPQEDMDVALMEHSNCSSEPGFWAHPPGAQ). The remaining sequence of TRPM4 is still of rat origin. Genotyping and DNA sequencing confirmed the presence of human TRPM4 sequence. All animal experiments were conducted in accordance with the guidelines of the Institutional Animal Care and Use Committee of the National Neuroscience Institute and international ARRIVE guideline (https://arriveguidelines.org). The experimental protocol was approved by the Institutional Animal Care and Use Committees (IACUC No. A19107). All rats were housed in Animal Research Facility, Lee Kong Chian School of Medicine, Nanyang Technological University.

### Primary culture of cortical neurons from transgenic rats

In brief, fetal brains from embryonic day 18 (E18) pregnant transgenic rats were obtained and digested for 40 min in Earle's Balanced Salt Solution (EBSS, Thermo Fisher Scientific, MA, USA) containing 20 U/ml papain (Worthington, Lakewood, NJ, USA). Dissociated cells were seeded on 12 mm round glass coverslips coated with poly-L-lysine and laminin with neuron culture medium containing Neurobasal Medium, 2% B27 supplement, 1% GlutaMAX supplement (Thermo Fisher Scientific, MA, USA). Medium was replaced 1 day after plating, and half of the medium was changed every 3 days. The cells were treated with 4 µM cytosine arabinoside from days in vitro (DIV) 3–6 to restrict mitotic cell proliferation and maintained for 10–21 days in neuron culture medium at 37 °C.

### In vitro hypoxic induction

For acute oxygen–glucose deprivation (OGD) during patch clamp recording, the cells were perfused with an anoxic artificial cerebrospinal fluid (aCSF) containing 5 mM NaN_3_ and 10 mM 2-deoxyglucose to reduce glycolytic capacity and interfere with cellular respiration in energy metabolism.

For 24-h OGD, the cells were grown in low glucose media (2 parts of EBSS mixed with 1 part of low glucose DMEM, Invitrogen, Life Technologies Corporation, USA) and placed in a polycarbonate hypoxia induction chamber (Modular Incubator Chamber, #27310, STEMCELL Technologies Inc. Vancouver, BC, Canada). The chamber was first flushed with a gas mixture containing 1% O_2_, 5% CO_2_ and 94% N_2_ for 5 min to purge the ambient air from the chamber. Following that, the hypoxia chamber was tightly sealed, and placed in a 37 °C incubator for 24 h.

### Immunofluorescence staining

Following fixation with 4% paraformaldehyde, cells was incubated in blocking serum (10% fetal bovine serum in 0.2% PBST) for 1 h. Primary antibodies include M4M (0.4 µg/ml), anti-Myc (C3956, Sigma-Aldrich, MI, USA), anti-WGA (Cat#W11261, Thermo Fisher Scientific, MA, USA), IgG from human serum (I4506, Sigma-Aldrich, MI, USA), anti-MAP2 (M4403, Sigma-Aldrich, MI, USA). Secondary antibodies are conjugated with FITC or Alexa Fluor 594, Alexa Fluor 647.

### Electrophysiology

Whole-cell patch clamp was used to compare the effect of M4M on human and mouse TRPM4. In brief, HEK293 cells were transfected with 1 μg human TRPM4^17^ or mouse TRPM4^15^ using lipofectamine 2000 transfection reagent (Cat#11668019, Thermo Fisher Scientific, MA, USA). 24 h after transfection, whole-cell currents were recorded at room temperature using a patch clamp amplifier (Multiclamp 700B equipped with Digidata 1440A, Molecular Devices, CA, USA). Patch electrodes were pulled using a Flaming/Brown micropipette puller (P-1000, Sutter Instrument, CA, USA) and polished with a microforge (MF200, World Precision Instruments Inc. FL, USA). The bath solution contained (in millimole/litre) NaCl 140, CaCl_2_ 2, KCl 2, MgCl_2_ 1, glucose 20, and HEPES 20 at pH 7.4. The internal solution contained (in millimole/litre) CsCl 156, MgCl_2_ 1, EGTA 10, and HEPES 10 at pH 7.2 adjusted with CsOH. Additional Ca^2+^ was added in the pipette solution to get 7.4 μM free Ca^2+^, calculated using WEBMAXC v2.10. Monoclonal antibody M4M, or mouse control IgG (subclass IgG1) was added into bath solution 30 min before recording at a concentration of 20.8 μg/ml. The current–voltage relations were measured by applying voltage ramps for 250 ms from − 80 to + 80 mV at a holding potential of 0 mV. The sampling rate was 20 kHz and the filter setting was 1 kHz. Data were analysed using pClamp10, version10.2 (Molecular Devices, CA, USA). Baseline currents were recorded on non-transfected HEK293 cells. Hypoxia was induced by applying a bath solution containing 5 mM NaN_3_ and 10 mM 2-deoxyglucose (2-DG) continuously through a MicroFil (34 Gauge, World Precision Instruments Inc. USA) around 10 μm away from the recording cells. The flow rate was set at 200 μL/min. Normoxic currents were recorded at 0 min and compared with hypoxic currents recorded 7 min after hypoxic incubation. Human and mouse TRPM4 currents can be obtained by subtracting baseline currents from non-transfected cells. Neuron recordings were performed in the presence of antagonists: 10 μM picrotoxin, 10 μM CNQX (6-cyano-7-nitoquinoxaline-2,3-dione), 50 μM D-APV (D(-)-2-amino-5-phosphonovaleric acid), 1 μM TTX (Tetrodotoxin), 60 μM cadmium and 100 μM 4AP (4-aminopyridine).

### Epitope substitution assay

To investigate the proposed epitopes and the target specificity of M4M, Epitope Substitution Scans were performed by PEPperPrint GmbH. The substitution scan of wild-type peptide ^1^SNCSSEPGFWAHP^13^ was based on an exchange of all amino acid positions with the 20 main amino acids. Peptides further away from binding epitope EPGF were not included due to the peptide length limitation of the assay. After on-chip peptide synthesis, all peptides were cyclized via a thioether linkage between a C-terminal cysteine and an appropriately modified N-terminus. The corresponding peptide microarrays contained 260 different peptide variants and were framed by additional HA (YPYDVPDYAG) control peptides. Microarrays were incubated with the M4M antibody at a concentration of 1 μg/ml for 16 h at 4 °C and orbital shaking at 140 rpm. Subsequent staining with secondary goat anti-mouse (H+L) DyLight680 antibody (Thermo Fisher) was followed by readout with a Innopsys InnoScan 710-IR Microarray Scanner and quantification of spot intensities, as well as peptide annotation with PepSlide Analyzer (Sicasys GmbH).

### Human TRPM4 structure analysis

Coordinate files for the calcium-free (PDB ID: 6BQR) and calcium-bound human TRPM4 tetramers (PDB ID: 6BQV)^19^ were downloaded from the Protein Data Bank (PDB) (http://www.rcsb.org/)^27^. Channel pore radii for dots visualization were calculated using the program HOLE^28^ and a sample size of 0.225 Å. Electrostatic surface potentials were calculated with the APBS web suite^29^ using default parameters. Channel pores, electrostatic surfaces and protein structures were visualized using the PyMOL Molecular Graphics System, Version 1.8. 2015. Schrodinger, LLC.

### Statistical analysis

Data are expressed as the mean ± s.e.m. Statistical analyses were performed using GraphPad Prism version 6.0. Student’s t test was used to compare two means. *P* < 0.05 was considered significant.

## Data Availability

All resources are available from the corresponding author on reasonable request. The polypeptide generated and analysed during the current study are available in the Uniprot repository (https://www.ebi.ac.uk/swissprot/, SPIN ID number SPIN200024323).
